# Extending biostasis to pregnancy: defining an unexamined biomedical problem

**DOI:** 10.3389/frph.2026.1950341

**Published:** 2026-10-21

**Authors:** Carrie A. Radomski, Natasha Vita-More

**Affiliations:** 1 Biomedical Research and Longevity Society, Fort Lauderdale, FL, United States; 2 Biostasis Technologies, New York, NY, United States; 3 University of Advancing Technology, Tempe, AZ, United States; 4 Geneva College of Longevity Science, Geneva, Switzerland

**Keywords:** biostasis, cryobiology, embryo, fetus, human cryopreservation, pregnancy

## Abstract

Our review of the scientific literature identified no studies directly addressing the cryopreservation of a pregnant individual or the preservation of an embryo or fetus *in utero*, despite the routine use of cryopreservation for embryos, oocytes, and sperm in reproductive medicine and the enrollment of individuals of reproductive age in biostasis organizations. This paper defines this unexamined problem by synthesizing evidence from cryobiology, reproductive and developmental biology, clinical medicine, bioethics, and law. It develops a conceptual framework that treats the pregnant individual and conceptus as one physical body containing two biologically distinct systems, and distinguishes established evidence, testable hypotheses, and speculative scenarios. Two falsifiable hypotheses follow: that cryoprotectant delivery across the maternal–fetal interface is rate-limited under whole-body perfusion, and that the resulting maternal–fetal concentration gradient varies with gestational age. The paper analyzes four preservation scenarios, the clinical precedent of somatic support after maternal brain death, and the consent, surrogate decision-making, and jurisdictional questions these scenarios raise. Membership data from three biostasis organizations indicate that roughly one in eight members of organizations reporting age data is a woman of reproductive age. The paper does not propose clinical protocols or claim feasibility. It sets out a prioritized research agenda and recommendations for organizational policy in a previously unexplored area of biomedical research.

## Highlights

Conceptus: The embryo or fetus together with its extraembryonic structures, including the placenta and fetal membranes. The term is used in this article for the developing organism and its supporting tissues across gestation.

Extracorporeal membrane oxygenation (ECMO): A temporary life-support technique that circulates blood outside the body for oxygenation and carbon dioxide removal before returning it to the patient, thereby temporarily replacing the functions of the heart and lungs.

Gestational age (GA): The clinical measure of pregnancy duration, conventionally calculated from the first day of the pregnant individual's last menstrual period and expressed in completed weeks and days. Gestational age is approximately two weeks greater than post-fertilization age and is the standard convention used in obstetric practice.

*In vitro* fertilization (IVF): An assisted reproductive technology in which mature oocytes are fertilized by sperm outside the body under laboratory conditions, with resulting embryos cultured before transfer to the uterus or cryopreservation.

Standby, Stabilization, and Transport (SST): The sequence of procedures performed by biostasis organizations following legal death to reduce ischemic injury, stabilize the patient, and prepare for transport to a facility where cryoprotective perfusion and cryopreservation can be initiated.

Viability: The developmental stage at which a fetus may be capable of sustained survival outside the uterus with available medical support. Viability is not a fixed gestational threshold and depends on multiple factors, including gestational age, fetal condition, birth weight, and the availability and capabilities of neonatal care.

Cryoprotective agent (CPA): A substance used to reduce cryopreservation injury by limiting ice formation and managing osmotic effects during cooling and warming.

Vitrification: Conversion of a cryoprotectant-containing biological system into a glass-like, noncrystalline state during cooling, intended to avoid damaging ice formation.

## 1 Introduction

Advances in cryobiology have enabled the long-term preservation of biological materials ranging from cells and tissues to whole organs and organisms (1, 2). Within this broader field, the distinct areas of biostasis and cryopreservation represent advanced applications aimed at preserving complex living systems at low temperatures for potential future recovery (2–4). Biostasis is the extreme slowing of biological activity in an organism without causing death. In nature, some animals enter a state of biostasis to endure harsh environments by naturally altering their own metabolic activities to an undetectably low level (4). Biostasis as a deliberate intervention includes cryopreservation, in which biological cells are preserved at low temperatures in a vitrified state of stasis. Biostasis can also take the form of chemical preservation (3), or a combination of both.

In this article, biostasis refers to the use of cryobiological methods to preserve biological structure and viability by greatly reducing or suspending metabolic activity, with the aim of maintaining the possibility of future recovery or restoration (4).

While cryopreservation is routine for sperm, oocytes, and embryos and central to assisted reproductive technologies (1, 5), our scoping search identified no empirical studies, case reports, reviews, or policy analyses directly addressing cryopreservation of an intact pregnant individual or preservation of an embryo or fetus *in utero*. We searched PubMed, Scopus, and Google Scholar using combinations of terms related to pregnancy, cryopreservation, biostasis, cryonics, embryo, and fetus. For this analysis, ‘directly addressing’ refers to literature specifically considering preservation of an intact pregnancy or an embryo or fetus *in utero*, rather than established cryopreservation of preimplantation embryos or isolated placental tissue. Because this was a scoping rather than systematic review, this finding should be interpreted as an absence identified by the search rather than proof that no relevant publication exists. The result nevertheless identifies a substantial gap in both cryobiology and the biostasis discourse.

The omission of pregnancy from biostasis-related discussion is notable because individuals of reproductive age are already enrolled with biostasis organizations (Table 1). The lack of conceptual groundwork and the absence of reported pregnancy-concurrent biostasis cases leave unresolved questions concerning preservation priorities during pregnancy, consent, and medical decision-making.

**Table 1:** Membership by sex and age, Alcor Life Extension Foundation, Tomorrow Biostasis, and Cryonics Institute, September 2024.

Organization	Total members	Female, *n* (%)	Female aged 20 to 50, *n* (%)
Alcor Life Extension Foundation	1,448	258 (17.8)	Not reported
Tomorrow Biostasis	624	110 (17.6)	74 (11.9)
Cryonics Institute*	787	208 (26.4)	97 (12.3)
All three organizations	2,859	576 (20.1)	Not calculable
Tomorrow Biostasis and Cryonics Institute	1,411	318 (22.5)	171 (12.1)

Alcor did not report age by sex. *Cryonics Institute figures include fully funded members only. Data obtained directly from each organization with written permission: D. Cremeens, Alcor Life Extension Foundation (personal communication, September 23, 2024); A. Casali, Tomorrow Biostasis (personal communication, September 25, 2024); H. McCauley, Cryonics Institute (personal communication, September 28, 2024).

Membership data obtained directly from three biostasis organizations in September 2024, with written permission, indicate the size of the population to which this question may apply (Table 1). Across 2,859 members of the Alcor Life Extension Foundation, Tomorrow Biostasis, and the Cryonics Institute, 576 (20.1%) are women. Age data by sex were available for Tomorrow Biostasis and the Cryonics Institute: of their combined 1,411 members, 171 (12.1%) are women aged 20–50, a range that approximates reproductive age, and a further 34 female members are under 20. Alcor reported sex but not age by sex, so its corresponding reproductive-age proportion could not be calculated. Among the two organizations reporting age data by sex, roughly one in eight members is a woman aged 20–50. None of the three organizations collects data on pregnancy status or reproductive intentions. These figures show that pregnancy-concurrent cases are foreseeable within current membership, while the absence of reproductive-status data limits any estimate of how frequently such cases might arise.

The sections that follow review the relevant cryobiology and reproductive biology, set out a conceptual framework and two testable hypotheses, analyze the biological constraints and preservation scenarios specific to pregnancy (6, 7), and examine the ethical and legal questions these scenarios raise (8, 9), before proposing research priorities and recommendations for biostasis organizations.

## 2 Cryobiology and biostasis

The field of cryobiology is the scientific study of how living systems respond to low temperatures, particularly temperatures below the freezing point of water (1, 2). It examines the physical, chemical, and biological effects that occur when cells, tissues, or organisms are cooled, frozen, and later thawed. Cryobiology investigates phenomena such as ice formation, osmotic stress, cellular dehydration, membrane damage, and the protective role of cryoprotective agents (1, 2). Its research spans many biological scales—from single cells to tissues and small organisms—and provides the theoretical and experimental foundation for modern cryopreservation techniques (1, 2).

Cryobiology has already achieved widespread practical application in medicine and reproductive biology (1, 5). The cryopreservation of sperm, eggs, and embryos is now routine in assisted reproductive technologies such as *in vitro* fertilization (IVF) (1, 5). These procedures rely on decades of cryobiological research into vitrification (10), cryoprotectant toxicity, cooling rates, and thawing protocols that preserve cellular structure and viability. Similar principles are also applied in the preservation of blood cells, stem cells, and certain tissues used in transplantation and biomedical research. In this sense, cryobiology provides the scientific groundwork that makes controlled preservation of living biological material possible.

The concept of biostasis is related but broader in scope. Biostasis refers to the stabilization of biological systems by extreme slowing of metabolic activity, often through extremely low temperatures, with the goal of preserving structural and molecular integrity for extended periods (2, 4). The distinction is one of scope rather than substance: cryobiology is the scientific discipline investigating the mechanisms of low-temperature effects, while biostasis describes the intended outcome, a state in which biological change is effectively paused. Biostasis can involve cryogenic temperatures but may also include other preservation techniques that suspend biological deterioration.

The two intersect most clearly in technologies designed to preserve living material for future use, where cryobiological research has enabled the cryopreservation of reproductive cells and embryos with metabolic processes suspended for years while viability is maintained. Applying these same principles to whole organs or organisms introduces substantially greater technical challenges, including cryoprotectant toxicity, ischemia, perfusion, and fracture prevention. In reproductive cryopreservation the two fields are closely aligned, because the biological systems are relatively small and post-thaw viability has been demonstrated (1, 5). In whole-organ preservation, the first successful transplantation of a functional cryopreserved rat kidney represents an important advance (11); extending these methods to whole human organisms remains an unresolved scientific challenge (2, 3).

### 2.1 Clarification of human cryopreservation and of limitations

This section outlines the scientific foundations that motivate attempts at human cryopreservation and the biological and technical limitations that continue to constrain the field. Distinguishing what is well supported by cryobiological research from what remains unresolved is essential for framing the analysis that follows.

#### 2.1.1 Scientific foundations of human cryopreservation

Several established findings in cryobiology provide the scientific foundation for attempts at human cryopreservation.

First, low temperatures dramatically slow biochemical reactions and biological degradation. The rate of molecular damage decreases sharply as temperature drops. At liquid nitrogen temperatures (approximately −196 °C), diffusion and most chemical reactions are reduced to extremely low rates, which allows biological structures to remain stable for long periods (2). This principle underlies all cryopreservation technologies used in medicine and research. Second, vitrification can prevent ice crystal formation, which is one of the main causes of cellular injury during freezing. By introducing cryoprotective agents and cooling tissues sufficiently rapidly, water can transition into a glass-like solid rather than forming ice. Vitrification has been successfully applied to many biological systems, including embryos, oocytes, and tissues, and is now standard in reproductive cryobiology (1, 5). Vitrification of a whole mammalian organ followed by life-sustaining transplantation has been demonstrated in a rat kidney model (11), though not in humans. Preservation of neural structure is particularly relevant because memory and identity are widely believed to depend on the physical organization of neural circuits. Third, long-term cryogenic storage is physically stable. Biological materials stored in liquid nitrogen can remain preserved for decades without measurable degradation when temperature stability is maintained (2). This principle is routinely demonstrated in reproductive medicine and biobanking (1, 5).

These findings provide a scientific basis for efforts to preserve complex biological systems through cryogenic stabilization.

#### 2.1.2 Current limitations and unresolved challenges

Despite these foundations, the application of cryogenic preservation to whole human organisms remains limited by several major scientific and technical challenges. One major limitation is cryoprotectant toxicity (12). The high concentrations of cryoprotective agents required for vitrification can damage cells and tissues, particularly during perfusion of large organs. Another challenge is uniform cooling and warming of large biological systems. Small samples can be cooled and rewarmed relatively evenly, but whole organs and bodies present difficulties due to temperature gradients. Uneven thermal changes can produce mechanical stress, fracturing, or devitrification (2, 3). A further limitation is ischemic injury prior to preservation. In most human cryopreservation scenarios, some delay occurs between circulatory arrest and cryoprotectant perfusion, during which oxygen deprivation can cause cellular damage (13). Additionally, while many cells, embryos, and some smaller biological systems can survive cryopreservation, reversible recovery of complex mammalian organs and whole human organisms following cryogenic preservation has not yet been demonstrated reliably. Finally, pregnancy introduces additional unknowns because maternal physiology, placental function, and fetal development form an interdependent system for which no established cryopreservation protocol or documented *in utero* preservation framework appears to exist in the current literature (3, 14). Recent whole-organ work has shown that vitrified rat kidneys can be rewarmed by nanowarming, which uses magnetic nanoparticles and alternating magnetic fields to produce rapid, relatively uniform volumetric heating and thereby reduce devitrification and thermal-stress injury (11). Pregnancy would add another level of thermal complexity because maternal tissues, placenta, amniotic fluid, and fetal tissues differ in geometry, water content, perfusion, and thermal mass. Even if adequate cryoprotectant concentrations could be achieved, preservation would therefore require characterization of cooling and warming rates across these compartments rather than assuming that maternal thermal behavior predicts fetal thermal behavior.

#### 2.1.3 Concerns of memory preservation in the developing and full brain

Current neuroscience indicates that memory depends substantially on the physical and functional organization of neural systems. Whether preservation of neural structure alone would be sufficient to preserve memory-related information, and still more broadly personal identity, remains unresolved. If these structures can be preserved with sufficient fidelity, the informational basis of memory may also remain preserved.

Evidence from studies of the nematode *Caenorhabditis elegans* provides partial support for the preservation of neural function following cryogenic storage (15). Experiments demonstrated that worms capable of learned behaviors prior to cryopreservation retained those behaviors after revival. Because these responses depend on specific neural circuits, the findings suggest that neuronal structures associated with long-term memory can survive cryogenic preservation under certain conditions. These findings provide proof of principle that learned neural function can survive vitrification and revival in a living organism. This study was conducted by one of the present authors and has not been independently replicated. However, the nervous system of *C. elegans* comprises approximately 300 neurons, compared with roughly 86 billion in the human brain. The difference in scale and architecture means these results demonstrate possibility in a simple system rather than evidence bearing directly on mammalian brain preservation. A recent preprint has reported vitrification of animal and human brains with retention of ultrastructural integrity (16); as this work has not yet undergone peer review, its findings should be regarded as preliminary. Its authors, affiliated with 21st Century Medicine and the Alcor Life Extension Foundation, also report osmotic brain shrinkage after perfusion with the M22 vitrification solution and ultrastructural damage consistent with osmotic injury from incomplete cryoprotectant removal, mechanisms equally relevant to placental and fetal tissue.

These findings support the broader scientific premise that preservation of neural structure may preserve the informational basis of memory, although the application of this principle to complex mammalian brains remains an open research question.

## 3 Reproduction as a framework for biostasis

Reproduction provides one of the clearest biological contexts in which the preservation and suspension of developmental potential can be observed, both in nature and in medicine. Across many species, reproduction is not a continuous process but may be delayed, paused, or synchronized with environmental conditions in ways that preserve embryonic viability until circumstances are more favorable (4, 17). Reproduction is therefore central to this discussion in two senses. First, it is already the area of medicine in which reversible preservation of continuing life has achieved the greatest practical success. Second, it marks the limits of current preservation science: existing reproductive cryobiology is highly effective but does not extend to later developmental stages, and preserving an implanted embryo, a fetus, or an ongoing pregnancy is far more complex than preserving a blastocyst in a clinical setting (1, 5, 18).

Embryonic diapause is a reproductive strategy in which embryo implantation or further development is suspended until external conditions are more suitable for offspring survival. In mammals that exhibit diapause, the embryo remains viable but developmentally paused, allowing reproduction to be uncoupled from immediate implantation and birth. Although embryonic diapause is biologically distinct from cryopreservation, it is conceptually relevant because it demonstrates that development can persist through a period of suspended progression. This illustrates a broader biological principle: reproduction is compatible with temporary interruption, and viability need not depend on uninterrupted development (17).

Modern cryobiology extends this principle into deliberate technological preservation. In reproductive medicine, cryopreservation has enabled the long-term storage of sperm, oocytes, and embryos. This has transformed assisted reproductive technologies by allowing reproduction to be separated from immediate fertilization, implantation, and pregnancy. These practices demonstrate that reproductive potential can be preserved outside the body for extended periods and later reactivated under controlled conditions (1, 5).

Commercial *in vitro* fertilization (IVF) and embryo cryopreservation operate within a relatively narrow developmental window. Pregnancy involves an ongoing maternal–fetal relationship in which the developing organism is biologically dependent on the pregnant individual. Once implantation has occurred, preservation can no longer be understood as the storage of an isolated developmental entity alone. It becomes a question of whether one is attempting to preserve the embryo or fetus separately, or the pregnant individual and the intact maternal–fetal unit together.

Prior discussion of this issue has been limited. Minerva and Sandberg (18) considered the possible future cryopreservation of embryos and fetuses after removal from the uterus, framing it primarily as a reproductive and ethical question rather than as a problem of preserving an ongoing pregnancy. Their discussion is relevant because it recognizes that later developmental stages may eventually become objects of preservation, however it does not address cryopreservation of the pregnant individual or the technical and biological constraints of preserving pregnancy *in situ*. The sphere of reproduction serves not merely as background, but as the point at which established reproductive cryobiology reaches its conceptual boundary and the unexamined problem of pregnancy biostasis begins (18).

### 3.1 Conceptual framework for pregnancy cryopreservation

Because no direct empirical literature currently exists on cryopreservation of an intact human pregnancy, this paper does not advance a single unifying hypothesis. Instead, it develops a conceptual framework that integrates established evidence from cryobiology, reproductive biology, developmental biology, clinical medicine, bioethics, and law, and from which specific empirically testable hypotheses follow. The framework treats the pregnant individual-conceptus unit as a single physical body containing two biologically distinct systems that may respond differently to cryoprotectant exposure and cooling despite being subject to a single preservation procedure. This two-system framework distinguishes pregnancy biostasis from single-patient biostasis and organizes the technical, ethical, and legal analysis that follows. Within this framework, the placenta and amniotic fluid are treated as distinct compartments for cryoprotectant distribution and thermal behavior rather than as additional biological systems: the two systems are the pregnant individual and the embryo or fetus, and the placenta and amniotic fluid lie between them.

Within this framework, three levels of knowledge are distinguished: (1) established evidence, referring to findings demonstrated within existing fields such as reproductive cryobiology, vitrification, developmental biology, and somatic support during pregnancy; (2) testable hypotheses, referring to questions that can be investigated empirically, such as placental cryoprotectant distribution, gestational-stage-specific tissue responses, and maternal–fetal effects of cooling and vitrification; and (3) speculative scenarios, referring to possible applications of biostasis to pregnancy that have not been empirically demonstrated and are presented here to define the problem space rather than establish feasibility. This distinction is maintained throughout the analysis to avoid extrapolating established findings beyond the evidence available.

Two hypotheses follow directly from the dual-system framing and are testable in appropriately selected animal models using existing perfusion and analytical methods, with model selection requiring consideration of species-specific differences in placentation and gestational physiology, as discussed in Section 7.2.

Hypothesis 1. Cryoprotectant delivery across the maternal–fetal interface is rate-limited under standard whole-body perfusion conditions, such that fetal tissue cryoprotectant concentrations will be significantly lower than maternal tissue concentrations.
Hypothesis 2. Cryoprotectant penetration across the maternal–fetal interface varies systematically with gestational age.

Both hypotheses are falsifiable. Hypothesis 1 predicts a measurable maternal–fetal concentration gradient following perfusion; a finding of equivalent maternal and fetal tissue concentrations would disconfirm it under the tested conditions. Hypothesis 2 predicts a measurable association between gestational age and the magnitude of that gradient; a finding of no systematic stage-dependent variation would disconfirm it. Neither hypothesis has been tested, and both are presented as directions for empirical investigation rather than as established findings.

### 3.2 Organizational policies for cryopreservation of the pregnant individual

Biostasis enrollment frameworks generally focus on the individual member and may not explicitly address pregnancy-specific preservation decisions. Given the absence of validated pregnancy cryopreservation protocols, a policy-first approach is warranted. Organizational frameworks should define the ethical, legal, and scientific conditions under which inquiry may proceed rather than presuming the existence of validated clinical practices. In this context, policy is not an extension of established pregnancy protocols but a structure for responsible consideration of an unexamined biomedical domain.

A central requirement is to recognize that a pregnant individual and conceptus form one physically integrated unit containing two biologically distinct systems that may respond differently to cryoprotectants and cooling despite being subject to a single preservation procedure. This distinction introduces biological complexities beyond standard cryopreservation and requires policies that clarify how decisions are made when maternal and fetal interests may diverge (19). Informed consent therefore involves considerations beyond those associated with a single individual. The pregnant individual's documented wishes and, where applicable, the authority of a legally authorized representative should remain central, subject to jurisdiction-specific law. Biostasis organizations should also specify how those wishes are interpreted in relation to the developing embryo or fetus, which cannot exercise autonomous decision-making. This may include the role of advance directives and the handling of cases in which consent is incomplete or absent. Established bioethical principles, including respect for autonomy and nonmaleficence, provide a necessary foundation but require pregnancy-specific application (20). Given the lack of validated procedures, organizations should also define clear boundary conditions and explicitly acknowledge unknowns in cryobiology, developmental biology, and obstetrics, particularly regarding fetal viability and post-preservation developmental trajectories.

Consequently, no direct scientific evidence currently supports the feasibility of cryopreserving a pregnant individual and subsequently recovering both the pregnant individual and the developing embryo or fetus. Organizations should therefore avoid claims that such procedures are presently proven, clinically achievable, or supported by existing evidence.

Legal considerations further complicate this domain. The regulatory status of the fetus, standards for determination of death, and liability frameworks vary across jurisdictions, requiring organizations to adopt adaptable policies that remain compliant with medical and reproductive law. This includes clarifying custodial responsibility for preserved biological systems and defining governance over long-term storage and future decision-making (21). Finally, it is advisable that organizations set up formal systems for reviewing and guiding research in this area that includes the fields of cryobiology, obstetrics, neonatology, ethics, and law, with transparent criteria for study initiation and risk disclosure. Given the high degree of uncertainty, policies need to explicitly distinguish between evidence-based findings and speculative extrapolation.

In sum, organizational policy in this domain must precede practice. By defining the problem space, clarifying ethical and legal responsibilities, and establishing appropriate governance and safeguards, such policies provide the necessary foundation for responsible scientific inquiry into a previously unexplored intersection of cryobiology, biostasis, and human development.

### 3.3 Complications of pregnancy in cryopreservation

The cryopreservation of a pregnant individual introduces complications beyond standard biostasis because pregnancy involves a maternal and fetal system that are biologically distinct yet functionally interdependent through the placenta. Maternal circulation, metabolism, endocrine signaling, immune regulation, and fluid balance are dynamically altered during pregnancy and directly influence fetal development through continuous maternal–fetal exchange (22, 23). As a result, any interruption of perfusion, oxygenation, temperature regulation, or tissue integrity during cryopreservation would affect not only the pregnant individual but also the intrauterine environment on which fetal development depends.

In addition, cryopreservation relies on cryoprotective agents whose toxicity, osmotic effects, and tissue-specific limitations remain significant challenges even in the preservation of cells, tissues, and organs (24, 25). The implications of such agents for a developing fetus, particularly across different stages of gestation, are unknown. Evidence from embryo and oocyte cryopreservation further suggests that developmental systems may be sensitive to cryopreservation-related stress and cryoprotectant exposure, including potential epigenetic and developmental effects, although these findings cannot be directly extrapolated to an ongoing pregnancy (26).

For these reasons, pregnancy must be understood within cryopreservation not as an extension of single-patient biostasis, but as a uniquely complex biomedical condition involving two interdependent systems, stage-specific developmental vulnerability, and a high degree of scientific uncertainty.

## 4 Biological foundations for biostasis during pregnancy

Any consideration of cryopreservation in a pregnant individual must account not only for maternal physiology, but also for the developmental status, viability, and biological vulnerabilities of the embryo or fetus. Unlike postnatal patients, pregnancy introduces dynamic changes in metabolism, circulation, immunology, and endocrine regulation, as well as specialized anatomical structures that mediate maternal–fetal exchange (23, 27). These factors have direct implications for the feasibility, risks, and outcomes of cryopreservation and cannot be extrapolated from existing biostasis or cryobiology literature focused on non-pregnant individuals (24, 25).

For this reason, a clear understanding of prenatal development is a prerequisite for evaluating potential biostasis scenarios involving pregnancy. Human gestation proceeds through distinct developmental stages—germinal, embryonic, and fetal—each characterized by different biological priorities, degrees of structural integration, and sensitivities to environmental disruption (28). These stages provide a necessary framework for assessing how cryogenic intervention might differentially affect maternal and fetal systems, and for identifying stage-specific constraints relevant to cryopreservation. Unless otherwise specified, gestational age in this article refers to clinical gestational age calculated according to standard obstetric convention; developmental ages measured from fertilization are identified explicitly as post-fertilization age.

Human pregnancy is a dynamic and staged biological process characterized by continuous cellular differentiation, organogenesis, and physiological integration between the pregnant individual and the developing organism (29). For analytical purposes, prenatal development is commonly described in three overlapping stages: the germinal, embryonic, and fetal stages, each defined by distinct developmental processes with differing biological sensitivities and constraints (28).

The germinal stage encompasses approximately the first two weeks following fertilization. It begins with the formation of a zygote and includes rapid mitotic divisions as the conceptus travels through the fallopian tube and implants in the uterine lining. During this period, early cellular differentiation occurs and the foundational structures that will give rise to both the embryo and the placenta are established. Developmental viability at this stage is highly sensitive to environmental disruption (29).

The embryonic period extends through eight completed weeks after fertilization (approximately 10 weeks' gestational age) and is characterized by organogenesis and the emergence of major anatomical structures. During this interval, the neural tube forms, primordial features of the head and limbs become distinguishable, and early cardiac structures develop. This period is characterized by rapid morphological change and heightened vulnerability to developmental perturbation.

The fetal period begins after eight completed weeks post-fertilization (approximately 10 weeks' gestational age) and continues until birth. During this phase, the developing organism undergoes substantial growth, functional maturation of organ systems, and increasing physiological complexity.

The fetus exists within a highly integrated maternal–fetal system, supported by specialized structures including the amniotic sac, the umbilical cord, and the placenta, which collectively regulate nutrient exchange, waste removal, and immunological and hormonal signaling (27). These structures are essential to sustaining development and maintaining homeostasis throughout gestation.

Placental structure and transport function also change substantially across gestation. Early pregnancy is characterized by continuing villous development and remodeling of the maternal–placental interface, whereas later gestation involves a larger, more highly vascularized exchange organ with a progressively thinned diffusion barrier and altered transporter expression and metabolic activity (22, 27). These changes could affect both the direction and magnitude of cryoprotectant transfer. For this reason, Hypothesis 2 is intentionally two-sided: gestational maturation could either reduce a maternal–fetal concentration gradient by facilitating exchange or increase it through changes in fetal mass, vascular complexity, distribution volume, and diffusion distance.

A full-term human pregnancy is conventionally defined as approximately 40 weeks, or 280 days, although gestational duration varies (22). Preterm delivery may occur spontaneously or be medically indicated, and neonatal outcome depends strongly on gestational age and available care. Most ectopic pregnancies cannot develop to viability and can pose substantial risk to the pregnant individual (30). Advances in neonatal medicine have enabled survival of some extremely premature infants at approximately 22–23 weeks of gestation, although outcomes vary and intensive medical intervention is required (31).

These developmental stages and their associated biological dependencies are central to any consideration of biostasis involving pregnancy, as the feasibility, risks, and ethical implications of preservation are likely to differ substantially across gestational phases.

## 5 Biological and technical constraints of cryopreservation during pregnancy

The most significant complications in pregnancy cryopreservation arise from the interaction of gestational stage, maternal clinical status, and fetal condition at the time of preservation. These variables create distinct scenarios, each with different scientific, clinical, and ethical implications, and must be considered together rather than in isolation.

Gestational stage introduces critical differences in developmental sensitivity. Organogenesis is largely complete by the end of the embryonic period at approximately eight weeks post-fertilization (approximately 10 weeks’ gestational age). A fetus at approximately four months (second trimester) is therefore undergoing growth and functional maturation of established organ systems rather than initial tissue formation, though the central nervous system remains in an active phase of neuronal proliferation, migration, and synaptogenesis that continues into the third trimester.

At approximately eight months (third trimester) or near term, the fetus is structurally developed but remains physiologically dependent on maternal systems, and may be particularly sensitive to ischemia and exposure to cryoprotective agents. A full-term fetus approaches independent viability, yet still relies on maternal circulation at the time of preservation. Developmental biology demonstrates that susceptibility to environmental and physiological stress varies significantly across gestation, but there is no empirical evidence for how cryopreservation affects these stages (26, 28). The key issue is that developmental stage determines biological resilience and risk, and these parameters remain undefined in the context of biostasis.

Maternal clinical status at the time of preservation further complicates outcomes. In cases of a pregnant individual declared dead by neurologic criteria and maintained on somatic support, circulation may be sustained temporarily, but long-term physiological stability is uncertain. Following circulatory arrest, ischemic delay may introduce additional injury to maternal tissues and alter the intrauterine environment. In severely compromised patients, such as those experiencing trauma or critical illness, perfusion, oxygenation, and metabolic regulation may be highly unstable. Clinical evidence indicates that maternal physiological condition directly affects fetal viability and development, underscoring that maternal status determines the quality of preservation for both systems (19, 32).

Fetal condition at the time of preservation also introduces distinct complications. Scenarios may include a viable fetus with intact development, a compromised fetus experiencing hypoxia, distress, or developmental abnormality, or a non-viable or deceased fetus. These distinctions are critical because they determine whether preservation is occurring within a viable developmental system or one that is already compromised or non-viable, fundamentally altering the biological context and potential outcomes (20).

Awareness and timing of pregnancy further affect decision-making. Pregnancy may be known prior to cryopreservation, allowing for planning, consent considerations, and procedural awareness. Alternatively, pregnancy may be unknown and discovered during perfusion or surgical intervention, or not detected at all. These scenarios introduce decision-making under uncertainty, particularly when no prior consent or policy guidance specific to pregnancy exists, highlighting the importance of anticipatory governance in clinical and experimental contexts.

Finally, procedural interactions between maternal and fetal systems introduce additional uncertainty. Cryoprotective agents administered to the maternal system will likely reach fetal tissues, yet their toxicity, osmotic effects, and developmental impact are not understood in this context. Perfusion procedures may disrupt placental circulation, and differences between mature and developing tissues may result in unequal responses to cooling, vitrification, and rewarming. Cryobiological research demonstrates that even in single-organ systems, achieving uniform preservation remains a significant challenge, reinforcing that procedures designed for one organism may have unpredictable and unequal effects on two interconnected systems (24, 25). Taken together, these scenarios illustrate that the primary complications do not arise from a single factor, but from the intersection of developmental stage, maternal condition, and procedural intervention in a context for which no established scientific framework or body of evidence currently exists.

### 5.1 Preservation scenarios and options

Given the absence of validated protocols for cryopreservation during pregnancy, it is necessary to outline potential preservation scenarios. These are not proposed procedures but conceptual frameworks that clarify the range of clinical situations in which a biostasis organization might encounter a pregnant individual, and the distinct biological and ethical considerations each presents.

Scenario A: Preservation of the Intact Maternal–Fetal Unit. The most straightforward scenario from an organizational standpoint involves preserving the pregnant individual without separation of the fetus before cryopreservation. In this case, the pregnant individual and gestating organism are preserved together. This approach avoids the immediate surgical and medical risks of fetal extraction but introduces the compounded technical challenge of preserving two physiologically distinct systems simultaneously. Cryoprotectant perfusion designed for adult human tissues may produce unequal concentrations across the maternal–fetal interface, and placental transport together with fetal circulatory conditions may impede or alter delivery to fetal tissues (24, 27). Cryopreservation of post-delivery placental tissue is established in research and tissue banking (33), but no research addresses the cryobiological behavior of the intact placenta as a functioning organ during ongoing gestation or the response of the developing fetal vasculature to cryoprotectant concentrations optimized for adult tissues. This is the empirical content of Hypothesis 1. A persistent maternal–fetal concentration gradient would indicate that a protocol optimized for adult perfusion does not deliver equivalent cryoprotectant concentrations to fetal tissue and may leave fetal tissue at greater risk of ice formation during cooling.

Scenario B: Delivery or Extraction Prior to Maternal Cryopreservation. A second scenario involves the deliberate delivery or surgical extraction of the fetus prior to initiating biostasis stabilization procedures. If gestation has reached the periviable period, delivery may be considered when clinically indicated and consistent with the pregnant individual's wishes and applicable law. Viability is probabilistic rather than a fixed gestational boundary and depends on gestational age, fetal condition, birth weight, sex, antenatal corticosteroid exposure, active treatment decisions, and the capabilities of the treating center (7, 31). In cases where the pregnant individual has been declared brain dead, existing medical literature on prolonged maternal somatic support suggests that fetal survival following delivery is achievable in a meaningful proportion of cases, with outcomes improving as gestational age advances (14). Following delivery, the standard cryopreservation Standby, Stabilization, and Transport protocol could proceed theoretically with respect to the maternal patient alone (13). However, this scenario introduces ethical complexity regarding the decision to terminate maternal somatic support, the timing of delivery relative to fetal viability, and the legal status of decision-making authority in the absence of maternal consciousness and possible delay in cryopreservation to the detriment of the pregnant individual.

Scenario C: Early-Gestation Separation and Independent Preservation. At early, previable gestational ages, if the pregnant individual is brain dead or otherwise imminently terminal, separation of the conceptus before maternal cryopreservation can be considered conceptually as a distinct future strategy. No validated clinical protocol exists for retrieval and cryopreservation of a post-implantation embryo or early fetus in this context. Any subsequent preservation would require substantial adaptation beyond existing assisted reproductive technology methods and remains entirely unvalidated. Modern vitrification of preimplantation embryos is technically mature and has demonstrated reliable post-thaw survival and developmental viability (1, 5), but these methods cannot be assumed to extend to post-implantation embryos or fetuses. If an independent preservation approach were ever shown to be feasible, it could in principle allow maternal cryopreservation to proceed while separately addressing the conceptus. This scenario raises substantial ethical and legal questions concerning the status of the extracted embryo or fetus, consent for future disposition, and long-term custodianship (18).

Scenario D: Preservation Proceeding Without Prior Knowledge of Pregnancy. A final scenario that must be acknowledged is one in which pregnancy is not detected prior to or during initial cryopreservation stabilization. Pregnancy may be unknown to the patient's designated representative, or may not be apparent during external assessment. In early gestation, particularly in the germinal and early embryonic stages, there may be no observable physical indicators. This scenario is particularly significant because existing biostasis organizational policies do not include pregnancy-specific screening or response procedures (13). The consequences of proceeding with standard perfusion and cooldown in an undetected pregnancy are unknown but carry implications for both maternal preservation quality and the developing organism. This scenario underscores the importance of incorporating pregnancy status assessment into organizational standby and stabilization procedures and of developing policies that define how to proceed when pregnancy is discovered mid-case.

These four scenarios are not exhaustive and are not intended to serve as clinical guidance. They are a preliminary framework for identifying the distinct biological, procedural, and ethical circumstances that any future research and policy development must address. Table 2 compares the four scenarios across the status of supporting evidence, principal technical constraints, ethical and legal issues, and implications for organizational policy.

**Table 2:** Comparison of the four preservation scenarios described in section 5.1 by status of evidence, principal technical constraint, ethical and legal issues, and implication for organizational policy.

Scenario	Status of evidence	Principal technical constraint	Principal ethical and legal issues	Implication for organizational policy
A. Preservation of the intact maternal–fetal unit	Speculative. Maternal–fetal cryoprotectant distribution is untested (Hypotheses 1 and 2).	Unknown cryoprotectant delivery to fetal tissue across the maternal–fetal interface; unequal responses of maternal and fetal tissue to cooling.	Fetus preserved without independent consent; no established legal status for a preserved pregnancy.	Consent documents should state whether the member accepts preservation of an intact pregnancy.
B. Delivery or extraction before maternal cryopreservation	Established clinical precedent for delivery after maternal brain death with somatic support (14, 32, 34).	Delayed maternal stabilization; ischemic and reperfusion injury during prolonged somatic support.	Fetal survival weighed against maternal preservation quality; scope of surrogate authority.	Advance directives should set gestational age thresholds and a maximum duration of somatic support.
C. Early-gestation separation and independent preservation	Speculative. No validated protocol exists for post-implantation retrieval and cryopreservation.	Established embryo vitrification applies to pre-implantation embryos only.	Status and future disposition of the extracted embryo or fetus; abortion regulations may restrict or prohibit extraction.	Custodianship and disposition provisions; jurisdiction-specific legal review before any such option is offered.
D. Preservation without knowledge of pregnancy	Established that early pregnancy may lack physical indicators; effects of standard protocols on an undetected pregnancy are unknown.	None specific; occurs under current standard protocols by default.	No opportunity for consent regarding the pregnancy.	Pregnancy-specific preference documentation and pregnancy assessment at case initiation where clinically appropriate, legally permissible, and feasible.

Evidence classifications refer to the support for the specific pregnancy-biostasis scenario: established evidence may inform component processes or clinical analogues, but none of the four scenarios has been validated as a pregnancy cryopreservation procedure.

### 5.2 Brain death during pregnancy and somatic support

The intersection of brain death and pregnancy is not without medical precedent, and the literature offers the closest available clinical analogue to some scenarios a biostasis organization might encounter. In a systematic review of 35 reported cases in which brain death occurred during pregnancy, Dodaro et al. (14) found a mean gestational age at maternal brain death of approximately 20 weeks and a mean duration of somatic support of approximately seven weeks. Twenty-seven neonates (77%) were born alive, and live-birth rates rose with gestational age at maternal brain death, from 54.5% at 14 weeks to 19 weeks 6 days, to 91.7% at 20 weeks to 23 weeks 6 days, and 100% from 24 weeks onward.

Because the evidence base consists of a small number of published cases selected for somatic support, the 77% figure should be interpreted as a description of the reported literature rather than as a population-level probability of survival. The cases nevertheless demonstrate that sustained maternal physiological support can, under intensive medical management, permit continued fetal development after maternal brain death.

However, the clinical course of brain death during pregnancy is complicated by a range of acute medical instabilities, including circulatory instability, diabetes insipidus, panhypopituitarism, poikilothermia, metabolic dysfunction, acute respiratory distress syndrome, and susceptibility to bacterial infections (14, 32). These complications place the fetus at ongoing risk and may require emergency intervention, including cesarean delivery, before somatic support can be withdrawn. For a biostasis patient who is both brain dead and pregnant, these same instabilities would complicate the standby and stabilization phases of a cryopreservation case, potentially accelerating maternal physiological deterioration and limiting the window available for organized cryopreservation response.

Powner and Bernstein (34) reviewed reported cases of extended somatic support after maternal brain death and developed management recommendations, but their review does not establish a discrete gestational threshold at which prolonged support becomes beneficial. The longest case they reported began support at 15 weeks, which should not be treated as a 15-week biological cutoff. Decisions about continued somatic support would instead depend on gestational age, maternal physiologic stability, fetal condition, applicable law, and the pregnant individual's documented wishes. Their management analysis emphasizes maintenance of uterine and placental blood flow as a central objective for continued fetal maturation. This creates a direct tension for pregnancy-concurrent biostasis: interventions intended to optimize systemic and uteroplacental perfusion during somatic support may not align with the timing or physiologic priorities of subsequent neural-tissue preservation. The conflict should therefore be framed as a case-specific balance among uteroplacental perfusion, maternal physiologic stability, fetal condition, preservation timing, documented wishes, and applicable law rather than as a gestational-age cutoff.

### 5.3 Biostasis cryopreservation protocols and their limitations in pregnancy

A common operational framework described in human cryopreservation practice is Standby, Stabilization, and Transport (SST), which begins after legal pronouncement of death (13). In this framework, stabilization includes restoration or maintenance of mechanical circulation, active cooling, administration of selected medications, and preparation for transport and subsequent cryoprotective perfusion at a biostasis facility (13). Specific protocols vary by organization and case circumstances.

This framework is not readily compatible with a brain-dead pregnant individual maintained on somatic support. In jurisdictions applying the Uniform Determination of Death Act (21) or equivalent neurological criteria, brain death constitutes legal death, but somatic support may continue specifically to sustain gestation. The practical conflict is that the pregnancy may be supported for days or weeks while the biostasis objective generally favors timely stabilization after circulatory arrest. During that interval, the original brain injury, critical illness, and complications of prolonged intensive support may continue to affect tissue integrity. A pregnancy-concurrent case therefore falls outside the usual timing assumptions of SST and requires an explicit policy for balancing these competing objectives.

At early, previable gestational ages, the potential fetal benefit of prolonged somatic support is uncertain and must be weighed against maternal clinical stability and the pregnant individual's preservation goals (34). Separation of the conceptus before maternal cryopreservation remains only a speculative future possibility because no validated clinical protocol exists for retrieval and cryopreservation of a post-implantation embryo or early fetus. As gestation advances through the periviable period, the possibility of neonatal survival generally increases, although outcome remains dependent on fetal and treatment factors and the capabilities of the treating center; this may strengthen the rationale for considering continued somatic support when consistent with the patient's wishes and applicable law (7, 14, 31).

These scenarios highlight that the standard SST protocol requires pregnancy-specific adaptation and that organizations currently lack the policy infrastructure to govern these decisions.

### 5.4 ECMO in biostasis and potential application to pregnant individuals

Extracorporeal membrane oxygenation (ECMO) is an advanced cardiac and respiratory support technology that maintains oxygenated circulation outside the body, allowing compromised cardiopulmonary function to be bypassed (35). ECMO has been used clinically for decades. Extracorporeal circulation has also been reported in some biostasis operations, although its routine use, indications, and outcomes in this setting are not well characterized in peer-reviewed literature (36, 37). In principle, extracorporeal support can maintain perfusion and oxygenation during stabilization and may reduce ischemic time when appropriately deployed. Venoarterial (VA) ECMO provides both circulatory and respiratory support, whereas venovenous (VV) ECMO provides respiratory support but depends on native cardiac output for systemic and cerebral perfusion (38). The distinction is important here because the proposed use of extracorporeal support to maintain cerebral and systemic circulation after loss of effective cardiac output would require an arterial-return configuration rather than VV support alone.

Normothermic regional perfusion (NRP), used in donation after circulatory determination of death, provides a related clinical precedent for restoring oxygenated regional circulation through extracorporeal perfusion after circulatory arrest and before organ recovery. Although NRP is designed for organ preservation and transplantation rather than biostasis, it demonstrates the controlled use of extracorporeal circulation to limit ischemic injury and assess organ viability after circulatory death (39).

In the context of a pregnant individual, ECMO presents a theoretically relevant intervention because it can maintain maternal circulation and oxygenation independently of cardiac function. Following legal death and delivery of a viable fetus or following brain death in a patient for whom continued somatic support is no longer indicated, ECMO could be initiated to provide cerebral and systemic perfusion during stabilization and cooldown. This could reduce the ischemic interval that otherwise accumulates between cessation of cardiac function and the initiation of cryoprotective perfusion. However, ECMO carries its own risks in this context, including the potential for further reperfusion injury and the technical complexity of deploying this technology in field settings (40). During an ongoing pregnancy, any extracorporeal strategy would also need to account for pregnancy-specific hemodynamics, including aortocaval compression, preservation of uteroplacental flow, cannula position, and anticoagulation (41). These considerations make ECMO an analogy for circulatory support rather than an established pregnancy-biostasis protocol.

### 5.5 Reperfusion injury and ventilation-associated risks

A potential risk when mechanical circulation is initiated or intensified after an ischemic interval is reperfusion injury. This issue is relevant to pregnancy-concurrent biostasis cases only when tissues have experienced preceding ischemia; prolonged somatic support itself does not necessarily imply repeated ischemia and reperfusion.

Reperfusion injury refers to cellular damage that can occur when blood flow is restored to previously ischemic tissue (40). Reintroduction of oxygen can trigger inflammatory and oxidative processes, including reactive oxygen species generation, activation of complement pathways, and membrane injury. In brain tissue that has experienced a preceding ischemic interval, restoration or augmentation of perfusion through ECMO or other mechanical circulatory support may contribute to reperfusion-related injury. The relevance and magnitude of this mechanism depend on the cause of brain injury, duration of ischemia, and continuity of circulatory support (40).

For a biostasis patient whose preservation objective includes structural and molecular integrity of the brain, any reperfusion risk following a preceding ischemic interval should be considered when mechanical circulatory support is initiated. Prolonged critical illness and immobilization during mechanical ventilation are also associated with deep vein thrombosis and thromboembolic complications despite prophylaxis (42); pregnancy itself is a hypercoagulable state (23), which may further increase thrombotic risk. Hemodynamic instability, infection, and other complications of extended intensive care may further affect the biological condition at the time cryopreservation begins.

These considerations argue against a universal duration of somatic support based on gestational age alone. Organizational policy should instead require explicit consideration of the pregnant individual's documented wishes, fetal viability and condition, maternal physiologic stability, the expected effect of delay on preservation quality, and applicable law.

### 5.6 Conceptual early-gestation separation and preservation

For pregnant individuals at early, previable gestational stages, extraction and independent preservation of the conceptus can be considered only as a speculative alternative to *in utero* preservation. No validated clinical protocol exists for retrieving and cryopreserving a post-implantation embryo or early fetus for this purpose. Established embryo vitrification methods were developed for preimplantation embryos handled *in vitro* and cannot be assumed to extend to material retrieved after implantation. Embryo vitrification is a technically mature procedure with well-documented post-thaw survival and demonstrated viability following transfer (1, 5). The cryopreservation of cleavage-stage embryos and blastocysts has been routine in assisted reproduction for decades and has been progressively refined to minimize ice formation and maximize developmental outcomes (5).

Minerva and Sandberg (18) considered the theoretical possibility of cryopreserving embryos and early fetuses removed from the uterus as a future reproductive and ethical option. Their analysis helps define the conceptual problem but does not provide empirical evidence that post-implantation embryos or fetuses can be cryopreserved and recovered. Current validated reproductive cryopreservation therefore remains limited to preimplantation embryos.

The technical limitations become much greater once development has progressed beyond the preimplantation embryo. A structurally developed fetus presents biophysical barriers including tissue mass, vascular complexity, differential organ responses, and cryoprotectant toxicity at scale. No documented case of a structurally developed fetus being cryopreserved and revived exists in the published literature, and no research has demonstrated this in humans. Accordingly, validated embryo vitrification should not be extrapolated to post-implantation embryos or fetuses; whether any future preservation strategy could bridge that gap remains unknown.

## 6 Ethical and legal challenges in the absence of precedent

### 6.1 Informed consent and autonomy

The principle of informed consent is foundational to medical and experimental interventions, requiring that individuals receive sufficient information about risks, procedures, and alternatives to make voluntary and autonomous decisions (20). In the context of cryopreservation, informed consent is complicated by the speculative nature of revival technologies, uncertainty regarding long-term outcomes, and the paucity of validated clinical protocols. These complications are substantially amplified when the individual is pregnant at the time of preservation.

General work on reproductive rights and informed consent emphasizes the centrality of the pregnant individual's autonomy in pregnancy-related medical decisions (8). In a biostasis context, those decisions may also have consequences for the developing embryo or fetus. Generic advance directives and pre-enrollment consent documents may not anticipate choices concerning prolonged somatic support, gestational-age considerations, delivery or extraction, or the future disposition of an embryo or fetus. This gap supports development of pregnancy-specific preference documentation rather than assumptions made after loss of decision-making capacity.

### 6.2 Pregnant individual-conceptus unit consent problem

A central ethical challenge in pregnancy cryopreservation that distinguishes it from standard biostasis cases is the absence of consent capacity in the developing fetus. While the pregnant individual's autonomous choices ordinarily remain central to medical decision-making, the scope of surrogate authority and the legal recognition accorded to fetal interests may vary according to jurisdiction and clinical circumstances (19).

The pregnant individual–conceptus unit has no direct precedent in existing biostasis practice and is only partially addressed in broader bioethical and reproductive-law literature. Beauchamp and Childress (20) identify nonmaleficence and respect for autonomy as core principles of biomedical ethics, but their application to pregnancy biostasis requires additional specification. Organizational policies should therefore state how the embryo or fetus is regarded for purposes of preservation decision-making—for example, as a dependent biological system within the maternal patient, a potential secondary patient where clinically and legally recognized, or a future entity with interests relevant to custodianship. The applicable framework may vary by jurisdiction and gestational stage.

#### 6.2.1 Fetal moral status and competing ethical frameworks

The moral status of the embryo or fetus remains one of the most contested questions in reproductive ethics. Philosophical accounts variously ground fetal moral status in the onset of morally relevant capacities, gradual development of those capacities, or the relationship between fetal interests and the autonomy of the pregnant individual (43–46). This paper does not attempt to resolve these competing positions. Rather, their relevance to pregnancy biostasis lies in the fact that different accounts of fetal moral status may produce different conclusions about preservation priorities, particularly as gestation progresses and independent viability becomes possible.

Pregnancy biostasis also creates the possibility of maternal–fetal conflict in an unusual form. An intervention intended to preserve fetal development—for example, prolonged somatic support to permit continued gestation, could delay cryopreservation and potentially compromise preservation of the pregnant individual's tissues, particularly the brain. Conversely, initiating biostasis rapidly to optimize maternal preservation could reduce or eliminate the possibility of continued fetal development. These competing interests should not be presumed to have a universal hierarchy. Where the pregnant individual's wishes are known, respect for reproductive autonomy and previously expressed preferences should remain central (19); where they are unknown, the ethical problem becomes substantially more difficult and requires explicit standards for surrogate decision-making.

#### 6.2.2 Surrogate decision-making in the absence of advance instructions

When the pregnant individual lacks decision-making capacity and has not provided pregnancy-specific advance instructions, a surrogate may be required to make decisions whose consequences extend to both the pregnant individual and fetus. The surrogate's role should be distinguished from independent representation of fetal interests. Established surrogate decision-making generally seeks to follow the patient's previously expressed wishes or, when these are unknown, to apply an appropriate best-interest standard. Pregnancy biostasis complicates this framework because a decision intended to benefit the fetus may conflict with the pregnant individual's previously expressed goals for timely and optimal preservation. A surrogate should therefore not automatically substitute personal beliefs about pregnancy or fetal moral status for the known values and preferences of the pregnant individual. Pregnancy-specific advance directives could substantially reduce this uncertainty by documenting preferences concerning continued somatic support, fetal delivery or extraction, gestational thresholds, and preservation priorities.

#### 6.2.3 Psychosocial considerations

Pregnancy-related biostasis could also present significant psychosocial challenges for families, surrogate decision-makers, and healthcare professionals. Decisions involving maternal brain death, prolonged somatic support, fetal viability, timing of delivery, or initiation of biostasis may occur under conditions of acute grief, uncertainty, and competing preservation interests (47). Family members or surrogates may be required to consider the previously expressed wishes of the pregnant individual while simultaneously confronting decisions concerning the developing fetus and the consequences of delaying or initiating preservation. Healthcare professionals may likewise face emotionally difficult and ethically unfamiliar circumstances when maternal and fetal interests cannot both be preserved optimally. Any future pregnancy-specific biostasis framework should therefore recognize the need for clear communication, psychosocial support, and appropriate counseling for those involved in decision-making (48). A fuller analysis of these issues will require engagement with the literature on bereavement, maternal critical care, and perinatal palliative care and represents an important area for future interdisciplinary research.

### 6.3 Pronatalism and decision-making integrity

As a normative concern rather than an empirical claim about any particular institution, a further ethical consideration involves the potential influence of pronatalist social values on the decision-making processes surrounding pregnancy cryopreservation. Pronatalism, broadly, is the cultural and institutional tendency to privilege reproduction and the continuation of pregnancy. Pronatalism may create implicit pressure on family members, institutional representatives, or biostasis organization personnel to prioritize fetal outcomes in ways that conflict with the biostasis patient's own expressed preferences or even act against the goal of optimal preservation. Ethical counseling and organizational governance in this domain must remain explicitly oriented toward the patient's documented wishes and must resist the substitution of external normative assumptions for genuine, informed consent (8).

### 6.4 Legal frameworks and jurisdictional variation

The legal landscape governing pregnancy, death determination, and biostasis practice varies substantially across jurisdictions, introducing significant complexity for organizations that operate across national and state boundaries. The Uniform Determination of Death Act (21) defines legal death in the United States as either irreversible cessation of circulatory and respiratory functions or irreversible cessation of all brain functions, but the application of brain death criteria in pregnant individuals maintained on somatic support is an area of ongoing legal and medical uncertainty. In some jurisdictions, laws governing the medical treatment of pregnant individuals impose obligations on healthcare providers that may conflict with the initiation of biostasis protocols following legal death.

The regulatory status of the fetus varies across jurisdictions and has been substantially affected by recent developments in reproductive law, including the overturning of established abortion-related precedents (9). Abortion regulation introduces an additional jurisdiction-specific consideration for pregnancy biostasis, particularly in scenarios involving separation or extraction of an embryo or previable fetus prior to maternal cryopreservation. In the United States, the legal characterization of such an intervention may depend on applicable state law, gestational age, fetal status, the purpose and method of the intervention, and statutory definitions of abortion and pregnancy termination. Following *Dobbs v. Jackson Women's Health Organization*, states differ substantially in the circumstances under which termination of pregnancy is permitted and in the scope of exceptions for threats to the life or health of the pregnant individual (49, 50). These questions may become particularly complex following maternal brain death, where the pregnant individual is legally dead but fetal circulation may continue through somatic support and legal questions may arise concerning pregnancy termination, continued physiologic support, and fetal interests (49, 50). The legal implications of extracting, cryopreserving, or otherwise handling fetal tissue in the context of biostasis remain undefined and will require jurisdiction-specific legal analysis. Accordingly, extraction or separation of an embryo or fetus for purposes associated with biostasis cannot be assumed to constitute a legally available procedure. Organizations are advised to seek specialized legal counsel before developing policies that govern pregnancy cases, and to ensure that any protocols adopted are compliant with applicable medical, reproductive, and tissue-handling laws in each relevant jurisdiction.

These differences can be illustrated by comparing the United States, European Union member states, and Switzerland across three dimensions. For determination of death, the Uniform Determination of Death Act recognizes irreversible cessation of all brain functions as death in the United States (21); in the European Union, determination of death is governed by national law, and international surveys have found substantial variation across countries in the legal basis, clinical criteria, and procedures for determining death by neurologic criteria (51, 52); Swiss law defines a person as dead when the functions of the brain, including the brainstem, have irreversibly ceased (53). For the legal status of the fetus, Swiss law recognizes legal personality from live birth and grants the unborn child legal capacity only on condition that it survives birth (54); the European Court of Human Rights has declined to decide in the abstract whether the unborn child is a person for the purposes of the right to life (55); and in the United States, fetal legal status varies increasingly by state following *Dobbs* (9, 49). For surrogate decision-making, a Swiss advance directive is binding on physicians unless it contains impermissible instructions or there is doubt that it reflects the patient's wishes (54), and the Council of Europe's Convention on Human Rights and Biomedicine requires that the previously expressed wishes of a patient be taken into account in ratifying states (56); in the United States, many states restrict the effect of advance directives during pregnancy (57). A pregnancy-specific biostasis directive that is binding in one of these jurisdictions may therefore have limited or no force in another.

Jurisdictional variation may also affect the extent to which fetal interests receive separate legal recognition. Depending on the jurisdiction and clinical circumstances, fetal interests may be addressed through statutory protections or other legal restrictions affecting the pregnant individual's advance directive or the authority of a surrogate decision-maker (19, 57). These distinctions become particularly consequential in pregnancy biostasis because decisions concerning somatic support, delivery, extraction, or initiation of cryopreservation could place maternal and fetal preservation interests in direct tension. Accordingly, organizations should not assume that a single consent or decision-making framework will apply across jurisdictions (57). Any future pregnancy-specific policy will require jurisdiction-sensitive legal review addressing maternal autonomy, fetal legal interests, surrogate authority, determination of death, and the legal status and disposition of embryos or fetuses following separation from the pregnant individual. Table 3 provides a comparative overview of selected legal considerations across the United States, Switzerland, and the European Union, illustrating how differences in death determination, pregnancy and fetal considerations, and decision-making frameworks may affect the application of pregnancy biostasis.

**Table 3:** Selected legal frameworks are summarized for comparison only. Their application to pregnancy biostasis remains uncertain and would require jurisdiction-specific legal review.

Jurisdiction	Death Determination	Pregnancy/Fetal Considerations	Decision-Making
United States (21, 49, 50, 57)	Circulatory or neurologic criteria; state implementation varies.	Fetal protections and pregnancy-related restrictions vary substantially by state.	Advance-directive and surrogate authority varies by state, including pregnancy-specific restrictions.
Switzerland (53, 54)	Death defined in federal transplantation law as irreversible cessation of brain function, including the brainstem.	Legal personality begins at live birth; the unborn child has legal capacity only if it survives birth.	Advance directive is binding unless it contains impermissible instructions or doubt exists about the patient's wishes; postmortem donation is separately regulated.
European Union/Council of Europe (51, 52, 55, 56)	Governed by national law; legal basis, clinical criteria, and procedures vary among member states.	The European Court of Human Rights has declined to decide whether the unborn child is a person under the right to life; fetal status is left to national law.	The Oviedo Convention requires that previously expressed wishes be taken into account in ratifying states.

## 7 Discussion

The analysis presented in this paper identifies several priorities for future scientific, ethical, and policy research. The research priorities outlined below address areas requiring investigation before any practical protocols for pregnancy cryopreservation can be responsibly developed.

### 7.1 Feasibility and research priority

Before proposing a research agenda, it is necessary to ask whether pregnancy biostasis warrants investigation at all given the unresolved state of the underlying field. Whole-body human cryopreservation has not been demonstrated to be reversible in any organism of comparable complexity, and reversible recovery of vitrified mammalian organs remains limited to isolated demonstrations such as the rat kidney transplantation model (11). Pregnancy biostasis therefore rests on a foundation that is itself unvalidated. A reasonable objection follows: if the single-patient case remains unsolved, investigating the substantially harder two-system case may represent a misallocation of limited research effort.

This objection has force and should be acknowledged directly. The hypotheses advanced in this paper are not offered as an argument that pregnancy biostasis is achievable, nor as a claim that it should be prioritized over foundational work in whole-organ preservation. Research effort in cryobiology is scarce, and the most consequential near-term advances are likely to come from organ preservation for transplantation, where clinical need is immediate and outcomes are measurable.

Two considerations nonetheless support limited investigation now rather than complete deferral. First, the question is not purely hypothetical: individuals of reproductive age are enrolled with biostasis organizations (58; Table 1), while no validated pregnancy-specific preservation protocol exists. A case could therefore arise before relevant evidence or consent frameworks are available, forcing decisions under time pressure. Defining the problem and documenting member preferences in advance require comparatively little technical investment.

Second, the hypotheses identified here are testable with existing perfusion and analytical methods and could generate knowledge relevant beyond biostasis. Measuring cryoprotectant distribution across the maternal–fetal interface could inform research on placental transport and low-temperature preservation of placental or reproductive tissues (33). The value of such experiments therefore does not depend entirely on eventual feasibility of whole-body biostasis.

The balanced position is that pregnancy biostasis does not warrant substantial diversion of cryobiological research effort at present, but that the policy and consent gap warrants immediate attention from organizations, and that the empirical hypotheses are worth pursuing opportunistically where they align with existing reproductive cryobiology programs. Claims of near-term feasibility are not supported by any evidence currently available and should not be made.

Resource allocation should follow the same dependency logic. Low-cost work such as policy development, consent design, retrospective literature synthesis, and *ex vivo* placental perfusion can precede resource-intensive pregnant-animal studies. Experiments should be favored when they can answer defined questions about placental transport, tissue cryoprotectant exposure, or thermal behavior that also have value for reproductive or organ-preservation research. Large-animal or non-human-primate studies would require substantially greater cost, specialized facilities, and ethical justification and should follow only if lower-complexity models establish a clear scientific rationale. In this sequence, pregnancy-specific research complements rather than displaces foundational work in whole-organ preservation.

### 7.2 Research direction and proposed guidelines

In the domain of cryobiology, the immediate research priority is direct testing of Hypotheses 1 and 2 in the conceptual framework. Testing Hypothesis 1 requires measurement of cryoprotectant concentrations in maternal and fetal tissues following controlled perfusion in a pregnant animal model, with maternal and fetal tissue concentrations, placental concentrations, and amniotic-fluid concentrations measured as predefined outcomes. Testing Hypothesis 2 requires the same measurements across defined gestational stages, with concentration relationships examined as a function of gestational age. These experiments use existing categories of perfusion and analytical methods, although protocol development, species selection, and safety constraints would require dedicated validation. Protocols should prespecify the cryoprotectant formulation and concentration ramp, perfusion pressure and duration, temperature, sampling sites, and analytical method. Depending on the cryoprotectant, concentration could be quantified by validated chromatographic or osmometric methods, while differential scanning calorimetry or other direct thermal assays could be used as secondary outcomes to assess ice formation and vitrification behavior. Rather than defining success only as a statistically lower fetal concentration, the more relevant physical endpoint is whether each sampled compartment reaches a concentration compatible with the vitrification conditions being tested.

Selection of animal models is particularly important because placental structure, maternal–fetal exchange, and gestational physiology vary substantially across mammalian species, and no single model fully reproduces human pregnancy (59, 60). Initial testing of maternal–fetal cryoprotectant distribution could be conducted in pregnant guinea pigs, whose hemomonochorial placenta resembles the human interhemal barrier, unlike the hemotrichorial placentas of mice and rats, and which have a relatively prolonged gestation and are established models of placental transfer and fetal development (60). Sheep provide a complementary large-animal model. Although ovine placentation is epitheliochorial and differs substantially from human placentation, the size of the fetus and placenta and the ability to catheterize maternal and fetal circulations permit controlled perfusion and repeated sampling, making sheep particularly useful for characterizing cryoprotectant concentration gradients, vascular distribution, and gestational-stage effects. Non-human primates provide the closest available approximation to human placentation and pregnancy physiology and could therefore serve as a later translational model if findings from other species justify further investigation (59, 60). However, their use would require substantially stronger scientific justification because of ethical, logistical, and cost considerations. A staged approach using complementary models would therefore be preferable to reliance on a single species, with results interpreted according to species-specific differences in placentation and maternal–fetal physiology.

Whole-organ reproductive cryopreservation also provides a methodological precedent for vascular perfusion, cryoprotectant delivery, and assessment of post-thaw tissue and vascular integrity. Research on whole-ovary cryopreservation has emphasized the difficulty of achieving uniform perfusion in a vascularized reproductive organ and the importance of preserving the vascular pedicle, while clinical fertility-preservation guidance continues to distinguish established ovarian-tissue cryopreservation from experimental vitrification approaches (61, 62). These studies do not demonstrate feasibility for pregnancy preservation, but they provide relevant methods and caution against assuming that successful perfusion of large reproductive tissues is uniform or biologically sufficient.

These measurements would establish whether cryoprotectant delivery across the maternal–fetal interface is rate-limited under the tested conditions and whether the magnitude of any gradient varies across gestation. Either outcome would be informative. If fetal tissue concentrations approach maternal concentrations, the principal technical obstacles would likely lie elsewhere. If a large and persistent gradient remains, protocols optimized for adult perfusion would not provide equivalent fetal cryoprotectant exposure under those conditions and could leave fetal tissue at greater risk of ice formation during cooling.

Existing placental tissue cryopreservation studies provide methodological starting points for tissue handling and cryoprotectant assay (33), though this work involves excised tissue explants and cannot substitute for measurement in an intact gestational system. The specialized architecture of the placenta, which continuously mediates nutrient exchange, waste removal, and hormonal signaling (27), introduces preservation challenges distinct from those studied in post-delivery fragments. Beyond the two hypotheses, the distribution, toxicity, and osmotic effects of cryoprotective agents across the maternal–fetal interface remain unexamined, as does the cryobiological behavior of placental tissue under vitrification conditions.

*Ex vivo* placental perfusion provides an intermediate model between isolated placental explants and whole-animal pregnancy. Dual-sided perfusion of human placental cotyledons is widely used to study maternal-to-fetal drug transfer, placental disposition, and vascular effects, and measured transfer often corresponds reasonably with *in vivo* observations, although protocols vary across laboratories (63, 64). Such models could be adapted initially to characterize cryoprotectant passage across the placental barrier, concentration-time profiles, tissue retention, and vascular effects before testing whole-pregnancy perfusion. Interpretation would need to account for the syncytiotrophoblast as the principal exchange interface, transporter-mediated movement, placental metabolism, and gestational changes in placental structure. Pharmacokinetic approaches developed for transplacental drug transfer could likewise inform sampling design and compartmental modeling, while not establishing that drug-transfer behavior will directly predict high-concentration cryoprotectant kinetics (63, 64).

In the domain of developmental biology, research is needed to characterize stage-specific vulnerability to cryobiological stressors across the embryonic and fetal periods, and to identify whether any gestational window presents more favorable biological conditions for *in utero* preservation than others (28, 29). The embryonic period, characterized by active organogenesis and heightened sensitivity to environmental disruption, is likely to present different preservation risks than the fetal period, during which organ systems are undergoing functional maturation within an increasingly complex maternal–fetal relationship. Placental development itself follows a distinct trajectory across gestation, with the early first trimester placenta being structurally and functionally different from the term placenta in ways that may affect cryoprotectant perfusion, vascular response, and tissue integrity under preservation conditions (27, 33). Defining these developmental and placental windows is a prerequisite for any future attempt to design gestational-stage-appropriate preservation protocols.

In the domain of clinical and organizational policy, biostasis organizations should develop pregnancy-specific provisions within their informed consent and advance directive frameworks. They should establish clear decision trees for managing pregnant individuals at different gestational ages, and create mechanisms for interdisciplinary oversight involving cryobiologists, obstetricians, neonatologists, ethicists, and legal counsel (20).

Consent and policy frameworks should also anticipate partial failure. A preservation attempt could, in principle, produce unequal outcomes—for example, better structural preservation of the pregnant individual than of fetal tissues, or the reverse. Because no validated recovery endpoint exists, such outcomes could create substantial uncertainty for surrogates, families, organizations, and future custodians and could intensify grief or conflict over disposition. Advance directives should therefore avoid implying that maternal and fetal preservation outcomes are inseparable and should state explicitly that preservation of one biological system would not guarantee preservation of the other.

Current membership data are insufficient to estimate how often pregnancy-concurrent cases might actually occur (Table 1). Organizations could therefore offer voluntary, privacy-protective mechanisms for members to document pregnancy-specific preservation preferences and, when operationally relevant, current pregnancy status, existing fertility-preservation arrangements, and designated decision-makers. Collection of reproductive information should be limited to what is necessary for care planning and governed by explicit privacy, access, and retention policies.

In cases of brain death with somatic support, where time permits a coordinated medical workup, pregnancy assessment can be incorporated through medical records review, direct confirmation from next of kin, and biochemical testing where clinically appropriate. In acute scenarios involving sudden legal death, where the standby and stabilization phases must proceed without delay, prospective screening is not feasible, and the case must rely on prior member documentation and next-of-kin disclosure. To support both scenarios, organizations should develop pregnancy-specific advance directive documents that allow members to articulate their preferences regarding gestational age thresholds, prioritization of maternal preservation versus fetal viability, willingness to undergo somatic support, and disposition of embryos in the event of early-pregnancy intervention.

## 8 Conclusion and implications for reproductive health

This paper establishes a foundational problem space for a previously unexamined biomedical concern: cryopreservation of a pregnant individual and the fate of the developing embryo or fetus in that context. Our scoping search identified no scientific literature directly addressing this scenario. The gap is not merely technical but spans biological, clinical, ethical, legal, and organizational questions that are tightly intertwined.

The priorities identified in this paper can be ordered by dependency because the answers to some questions determine whether others matter. The first research priority is testing Hypotheses 1 and 2, because cryoprotectant distribution across the maternal–fetal interface determines whether preservation of an intact pregnancy is technically coherent under a given perfusion strategy. If fetal tissue does not reach vitrification-adequate concentrations under tested maternal-perfusion conditions, Scenario A would not appear technically viable under those conditions, shifting attention toward alternative scenarios. The second priority is characterizing stage-specific vulnerability of embryonic and fetal tissue to cryoprotectant exposure and cooling. The third is examining how prolonged maternal somatic support affects the condition relevant to subsequent preservation, because that relationship informs the central trade-off in Scenario B. The fourth is jurisdiction-specific legal analysis of delivery and separation scenarios to determine which options could lawfully be incorporated into organizational policy.

Policy development does not need to wait for these empirical studies. In the near term, biostasis organizations should consider pregnancy-specific provisions in enrollment documents and advance directives, including preferences concerning somatic support, maternal preservation priorities, gestational-age considerations, designated decision-makers, and disposition of any separated embryo or fetus. Organizations should also state plainly that no validated procedure exists for preserving an intact human pregnancy. They can additionally develop privacy-protective procedures for documenting pregnancy-specific preferences and for assessing pregnancy status at case initiation where clinically appropriate, legally permissible, and feasible. Over a longer horizon, empirical results bearing on Hypotheses 1 and 2 can inform whether pregnancy-specific preservation protocols merit further development.

This paper does not propose clinical protocols, nor does it assert that any form of pregnancy cryopreservation is currently feasible or advisable. Its purpose is to articulate the problem clearly enough for responsible scientific and policy inquiry. With roughly one in eight members of the two organizations reporting age-by-sex data being a woman aged 20–50 (Table 1), pregnancy-concurrent cases are a foreseeable organizational contingency rather than a purely theoretical question.

## Data Availability

The original contributions presented in the study are included in the article. The aggregated membership data summarized in Table 1 were supplied directly by the three organizations with written permission. Further inquiries can be directed to the corresponding authors.
